# Estimating marine survival of Atlantic salmon using an inverse matrix approach

**DOI:** 10.1371/journal.pone.0232407

**Published:** 2020-05-19

**Authors:** Sebastián A. Pardo, Jeffrey A. Hutchings

**Affiliations:** Department of Biology, Dalhousie University, Halifax, NS, Canada; Department of Agriculture, Water and the Environment, AUSTRALIA

## Abstract

The marine phase of anadromous Atlantic salmon (*Salmo salar*) is the least known yet one of the most crucial with regards to population persistence. Recently, declines in many salmon populations in eastern Canada have been attributed to changes in the conditions at sea, thus reducing their survival. However, marine survival estimates are difficult to obtain given that many individuals spend multiple winters in the ocean before returning to freshwater to spawn; therefore, multiple parameters need to be estimated. We develop a model that uses an age-structured projection matrix which, coupled with yearly smolt and return abundance estimates, allows us to resample a distribution of matrices weighted by how close the resulting return estimates match the simulated returns, using a sample-importance-resampling algorithm. We test this model by simulating a simple time series of salmon abundances, and generate six different scenarios of varying salmon life histories where we simulate data for one-sea-winter (1SW)-dominated and non-1SW dominated populations, as well as scenarios where the proportion returning as 1SW is stable or highly variable. We find that our model provides reasonable estimates of marine survival for the first year at sea (*S*_1_), but highly uncertain estimates of proportion returning as 1SW (*P*_*r*_) and survival in the second year at sea (*S*_2_). Our exploration of variable scenarios suggests the model is able to detect temporal trends in *S*_1_ for populations that have a considerable 1SW component in the returns; the ability of the model to detect trends in *S*_1_ diminishes as the proportion of two-sea-winter fish increases. Variability in the annual proportion of fish returning as 1SW does not seem to impact model accuracy. Our approach provides an instructive stepping-stone towards a model that can be applied to empirical abundance estimates of Atlantic salmon, and anadromous fishes in general, and therefore improve our knowledge of the marine phase of their life cycles as well as examining spatial and temporal trends in their variability.

## Introduction

Studies on population dynamics and life histories depend critically on age- or stage-specific estimates of natural mortality [1, 2]. Despite their necessity in predicting or hindcasting spatio-temporal trends in population size, mortality often represents the fundamental parameter about which the least is reliably known. The challenge is particularly acute for organisms that spend an extended period of time in the ocean, an environment that presents considerable logistical challenges for estimating survival relative to most terrestrial and riverine counterparts.

The Atlantic salmon (*Salmo salar*) is one of few vertebrates that spends substantive parts of its life cycle in both fresh water and the sea. Following the autumn spawning period in rivers, the young hatch in early spring and typically spend 1-4 years in fresh water before migrating to sea as smolts. After usually one to three winters at sea, adults return to their natal fresh water environment to spawn. Atlantic salmon are capable of migrating to and from the ocean more than once, spawning again as ‘returning spawners’ every time they return to fresh water.

In terms of mortality, the initial 1 to 6 months at sea, i.e., the ‘post-smolt’ period, is considered to be the period during which the daily per capita mortality experienced at sea is greatest [3]. Thus, the marine phase of anadromous Atlantic salmon is often considered the most important in relation to their overall population dynamics [4]. Recent declines in abundance of North American Atlantic salmon populations have been attributed to reductions in their marine survival [5, 6].

While there are multiple hypotheses of the underlying causes of this reduction in marine survival (e.g., increased temperature, change in oceanic conditions, etc.), very little is known about the spatio-temporal dynamics of Atlantic salmon survival at sea [5]. Changes in marine survival have the potential to drive changes in life histories, for example by compensation through selective survival or fisheries-induced evolution, and increases in marine survival can potentially result in Atlantic salmon populations having lower sea ages at maturity [7, 8]. Furthermore, recent research on Atlantic salmon has shown that sea age and repeat spawning are genetically correlated [9], which can potentially further reduce the reproductive capacity of individual spawners [10]. Thus, reliable estimates of marine survival are crucial to better understand the ecology and dynamics of this species.

Estimating mortality for marine fishes is difficult, and the complex iteroparous life cycle of anadromous Atlantic salmon makes it particularly challenging to accurately estimate marine survival for this species [11, 12]. When smolt abundance estimates are not available, marine survival has been calculated using aggregated data covering broad ocean basins, allowing for marine survival to be estimated indirectly using stock-recruitment (S-R) relationships [13, 14]. Recent evidence suggests that most mortality at sea occurs during the early migration (i.e., post-smolt) stages [3], meaning that population-level differences in post-smolt survival cannot be accounted for by basin-level assessments. Furthermore, marine survival estimates from S-R relationships do not account for population-level differences in egg-to-smolt survival. Incorporating smolt data would be beneficial for estimating marine survival, as it would remove the need to model S-R relationships.

When smolt abundance estimates are available, marine survival can be approximated by estimating return rates, which is the ratio in the number of adult returns and out-migrating smolts from the previous year. For Atlantic salmon populations that only spend one winter at sea (a ‘one-sea-winter’, or 1SW, salmon is also called a ‘grilse’) return rates are indicative of marine survival rates as most returning adults are from the same smolt cohort. However, these rates can be misleading for two-sea-winter (2SW) or other multi-sea-winter populations, given the confounding effect of the complex Atlantic salmon life history [12]. Thus, when salmon in a given population return to their natal streams to spawn after more than one year at sea, marine survival encompasses multiple years at sea. As a result, marine survival for these populations is often estimated using a maturity schedule model, also known as Murphy’s method which, at a minimum, allows for estimating survival in the first and second years at sea as well as the proportion of fish that return after one year at sea [15–17]. However, estimating both survival in the second year at sea and proportion returning after a year at sea is not mathematically possible without separating male and female salmon returns and assuming their survival rates are the same [17–19]. This is problematic, as traditional methods of sex determination are inaccurate [20] and Murphy’s method is very sensitive to biases in the sex ratio of smolts [19].

Matrix models can be useful for estimating demographic parameters. They have unique properties that can provide additional information that is of biological relevance [1]. Normally, matrix models use known life-history parameters to estimate population trends. However, matrix models can also be used to estimate life-history parameters when population trends are known; this is known as an inverse matrix analysis [1]. Inverse matrix approaches can be useful for estimating age- or stage-specific life-history parameters for populations for which there are time series data available [21–23].

Here, we develop a simple maturity schedule model that uses a simplified stage-structured matrix, without separating data between sexes, to estimate annual marine survival in the first and second years at sea (*S*_1_ and *S*_2_), as well as the proportion of fish returning after one winter at sea (*P*_*r*_), using a sample-importance-resampling algorithm, which approximates posterior distributions for problems where multiple parameters have to be estimated at once (also known as high dimensional problems, [24]). We also assess the accuracy and precision of the model by generating a simulated salmon abundance time series with known annual values of *S*_1_, *S*_2_, and *P*_*r*_. In particular, we aim to identify whether a simplified maturity schedule model is able to accurately estimate marine survival in the first winter at sea (*S*_1_).

## Methods

### Data simulation

The performance of the models developed in the present study were evaluated using a simulated Atlantic salmon abundance time series with six slightly different scenarios of varying life histories (Table 1). We create a 20-year time series with pre-set numbers of smolts every year (10,000), account for observation error, and calculate the numbers of 1SW and 2SW returns in subsequent years, using set values of *S*_1_, *S*_2_, and *P*_*r*_ from Table 1. We varied the value of *S*_1_ across years by gradually increasing it from 0.02 to 0.20 during the 20-year period; to simplify the assessment of whether the model extracts any information of the other parameters, we used fixed values of *S*_2_ (0.4) across years. We assumed salmon abundances were continuous rather than discrete values (i.e., fractions instead of integers) to allow the use of continuous probability distributions.

**Table 1:** Parameters used to simulate time series data of Atlantic salmon in the six scenarios.

	Scenario 1	Scenario 2	Scenario 3	Scenario 4	Scenario 5	Scenario 6
Parameter	Fixed *P*_*r*_, 1SW-dominated	Variable *P*_*r*_, 1SW-dominated	Fixed *P*_*r*_, mixed 1SW-2SW	Variable *P*_*r*_, mixed 1SW-2SW	Fixed *P*_*r*_, 2SW-dominated	Variable *P*_*r*_, 2SW-dominated
*S*_1_	*seq*(0.02, 0.2)	*seq*(0.02, 0.2)	*seq*(0.02, 0.2)	*seq*(0.02, 0.2)	*seq*(0.02, 0.2)	*seq*(0.02, 0.2)
*S*_2_	0.4	0.4	0.4	0.4	0.4	0.4
*P*_*r*_	0.95	*unif*(0.6, 0.95)	0.4	*unif*(0.2, 0.7)	0.15	*unif*(0.05, 0.3)

We ran scenarios with three different life history strategies: 1SW-dominated (relatively high *P*_*r*_), 2SW-dominated (relatively low *P*_*r*_) and a mixed 1SW-2SW scenario (intermediate *P*_*r*_). For each life-history scenario, we used either a fixed *P*_*r*_ value or a variable *P*_*r*_ (i.e., drawn from a distribution) for a total of six scenarios (Table 1). We removed the first three years from any simulation output because these years have incomplete abundance values for 2SW returns.

We first simulate a stable run of 10,000 smolts for every year and include observation error in these estimates

$$smolts_{t}=10000+\epsilon_{smolts,t}$$ (1)

where *ϵ*_*smolts*,*t*_ is the error term and is defined as *ϵ*_*smolts*,*t*_ = *Normal*(0, 500), which is equivalent to a coefficient of variation of 5%. We then simulate the smolt-to-returning adult part of the life cycle using the following maturity schedule equations:

$$R_{1,t}=smolts_{t-1}*S1_{t}*Pr_{t}+\epsilon_{1,t}$$ (2)

$$R_{2,t+1}=smolts_{t-1}*S1_{t}*\left(1-Pr_{t}\right)*S2_{t+1}+\epsilon_{2,t}$$ (3)

where *R*_1,*t*_ and *R*_2,*t*+1_ are the simulated abundances of 1SW and 2SW salmon returning in years *t* and *t*+ 1, respectively, *smolts*_*t*−1_ is the number of outmigrating smolts in year *t* − 1, *S*1_*t*_ is the proportion of salmon surviving in their first year (*t*) at sea, *Pr*_*t*_ is the proportion of salmon that return to spawn at year *t*, *S*2_*t*+1_ is the survival in their second year at sea of the same cohort of salmon who did not return to spawn at year *t*, and *ϵ*_*t*_ is the observation error term. This error term is heteroscedastic and is modeled as *ϵ*_*s*, *t*_ = *Normal*(0, *R*_*s*,*t*_ * 0.05), with *s* being the life stage (1SW or 2SW), and *t* being the year.

For simplicity, and to focus on assessing the estimation of marine survival in the first winter at sea, we do not model repeat spawners and we assume that no fish spend three or more winters at sea before returning to spawn for the first time. This assumption is representative of many populations in southeastern Canada, where maiden spawners spending more than two winters at sea are rare. Nonetheless, modeling only 1SW and 2SW returns results in an identifiability issue between *S*_2_ and *P*_*r*_, where these two parameters cannot be estimated independently [19].

### Marine survival estimation

We estimate marine survival on our simulated dataset using a maturity schedule model, which allows for estimating survival in the first and second years at sea as well as the proportion of fish that return after one year at sea [15–17]. We first model the smolt-to-return part of the life cycle using the maturity schedule model outlined in Eqs 2 and 3. These maturity schedule equations are embedded in a simplified 3 × 3 stage-structured matrix that only includes information on the abundance of smolts, 1SW, and 2SW returns:

$$\left(\begin{array}{ccc} 0 & 0 & 0\\ Pr_{t}\cdot S_{1,t} & 0 & 0\\ \left(1-Pr_{t}\right)\cdot S_{1,t}\cdot S_{2,t+1} & 0 & 0 \end{array}\right)\times \left(\begin{array}{c} smolts_{t-1}\\ 0\\ 0 \end{array}\right)=\left(\begin{array}{c} 0\\ 1SW_{t}\\ 2SW_{t+1} \end{array}\right)$$ (4)

This projection matrix is not a Leslie matrix as it can only estimate abundance of 1SW and 2SW returns for separate years in the future. In accordance with empirical data for most salmon populations [25], we assume there are no maiden fish that spend three or more winters at sea. We also assume that the survival probability of the second year at sea (i.e. *S*_2_) is additive to the survival of the first year at sea (*S*_1_), even though the fish that remain at sea after the first winter do not return to the estuaries like 1SW fish do.

### Sample-importance-resampling model

We use a sample-importance-resampling (SIR) algorithm to approximate the distribution of survival estimates for *S*_1_, *S*_2_, and *P*_*r*_ [24]. In a nutshell, the SIR method consists of 1) generating a distribution of matrices based on prior parameter distributions, 2) calculating the likelihood of each matrix in the distribution based on how well the resulting estimated returns match the simulated returns, and 3) resampling the distribution of matrices weighted by the likelihood to obtain pseudo-posterior parameter distributions [24].

We first generate a distribution of matrices by drawing 30,000 parameter vectors. The parameters used to populate Eq 4 are drawn from the following distributions:

$$\begin{array}{l} S_{1,t}∼exp\left(-lognormal\left(0.6,0.3\right)\right)\\ S_{2,t}∼exp\left(-lognormal\left(0.2,0.3\right)\right)\\ Pr_{t}∼\{\begin{array}{ll} inv\_logit\left(logistic\left(2.4,0.35\right)\right) & \text{For}\;1\text{SW}-\text{dominated}\;\text{populations}\\ inv\_logit\left(logistic\left(-0.5,0.5\right)\right) & \text{For}\;\text{Mixed}\;1\text{SW}-2\text{SW}\;\text{populations}\\ inv\_logit\left(logistic\left(-1,0.5\right)\right) & \text{For}\;2\text{SW}-\text{dominated}\;\text{populations} \end{array} \end{array}$$ (5)

Note that the priors for *S*_1_ and *S*_2_ are based on log-normal priors for the instantaneous mortality rates (*Z*_1_ and *Z*_2_) and are converted back to survival by *S* = *e*^−*Z*^. Similarly, the prior of *P*_*r*_ is converted from logit-transformed *P*_*r*_, so that *logit*(*Pr*_*g*_)∼*logistic*(*μ*, *s*). Assessing the proportions 1SW and 2SW returns is straightforward to do by looking at the yearly abundance estimates, and thus can help inform the priors being used, hence our different choices of priors for *P*_*r*_.

We then multiply the matrix with the population vector for year *t*, which is based on simulated abundances of smolts and adult returns (see Eq 4), to obtain the estimated population vector for year *t* + 1. The values from previous years (specifically *S*_1,*t*−1_ and *Pr*_*t*−1_) were included by estimating a median parameter value from the sample-importance-resampling routine in the preceding year, which means uncertainty in those parameters was not propagated. The population vector at each year, specifically the estimates of 1SW and 2SW, are our estimated returns that are used in the likelihood function below.

#### Likelihood function

We drew 10,000 samples from the joint prior (with replacement) by weighting the resampling based on the likelihood for every set of parameters in each year year *t* based on the difference between the estimated and simulated returns for each life stage *i* (in this case 1SW and 2SW). The likelihood function is based on the algorithms used in Wilson et al. [26], Brandon et al. [22], Zhang [27], and Smart et al. [23]. We used the following equation

$$L\left[D|\theta_{t}\right]=exp-\sum_{i=1}^{2}\frac{{\left(R_{i,t}^{obs}-R_{i,t}^{est}\right)}^{2}}{C*\sigma_{i}^{obs}}$$ (6)

where $R_{i,j}^{obs}$ and $R_{i,j}^{est}$ are simulated and estimated returns, respectively, for years *t* and life stages *i* (1SW and 2SW), and $\sigma_{i}^{obs}$ is the standard deviation of simulated abundances of life stage *i*. The constant *C* controls the “spread” of the likelihood function, where higher values result in wider distributions. We considered the SIR algorithm to have converged when the number of unique parameter vectors in the sample from the posterior is higher than 50% of the total number of parameters in the prior (i.e. >3000) and when no point in the posterior is assigned more than 1% of the total probability [24]. The value of *C* was adjusted for each scenario so that the SIR algorithm resulted in converged posteriors.

The cost function is scaled by the variance of simulated abundance estimates for each life stage. This likelihood function is applied to the returns estimates produced from a given matrix in a given year (Eq 4), thus each matrix in the distribution of parameters has an associated cost value in a given year. Finally, the resulting likelihood values were used to weight the resampling of matrices with replacement, with a weighting equivalent to the likelihood. The resulting distribution of resampled matrices is the one that produces the final estimates of *S*_1_, *S*_2_, and *P*_*r*_ for each year of the six scenarios. All models were written in R v3.6.1 [28].

## Results

**Fig 1:**
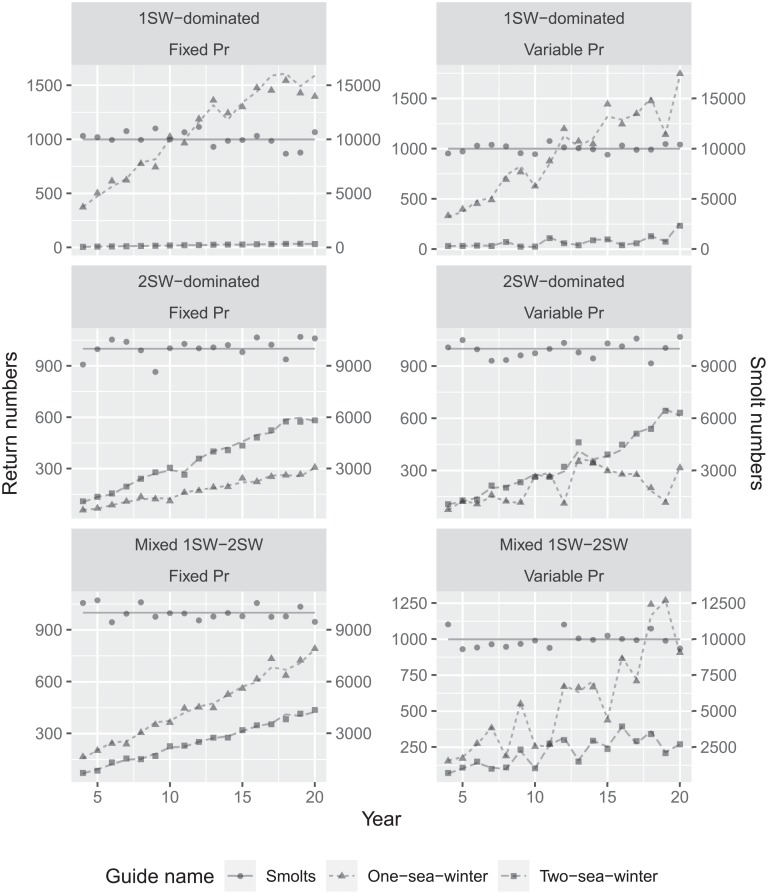
Simulated time series of returning adult salmon abundance in the six scenarios. The lines denote the simulated abundance estimates without observation error while the points are the same estimates including observation error.

The simulated time-series of salmon returns resulted in increasing salmon abundances in all six scenarios (Fig 1), with the highest increases in abundances correlated to scenarios with higher *P*_*r*_ values and, thus, those with more 1SW returns. The 1SW-dominated scenarios resulted in almost no 2SW returns, while in the mixed 1SW-2SW scenarios 1SW returns were slightly more numerous than 2SW returns, the latter two being similar in their simulated abundances (Table 2). Returns of 2SW were more abundant than 1SW returns only in the 2SW-dominated scenario.

**Table 2:** Simulated abundance time series of smolts, one-sea-winter (1SW), and two-sea-winter (2SW) returns for all six scenarios examined.

	Scenario 1			Scenario 2			Scenario 3			Scenario 4			Scenario 5			Scenario 6		
Year	Smolts	1SW	2SW	Smolts	1SW	2SW	Smolts	1SW	2SW	Smolts	1SW	2SW	Smolts	1SW	2SW	Smolts	1SW	2SW
1	10685	-	-	9478.4	-	-	10283.8	-	-	8999.5	-	-	10436.4	-	-	10373.4	-	-
2	9718	199.9	-	9954.9	147.0	-	9753.6	82.1	-	10166.9	90.0	-	10484.8	32.6	-	9787.2	27.6	-
3	10182	247.9	4.3	10311.8	213.0	20.8	10000.0	106.1	48.8	10585.7	128.1	35.3	10191.9	42.5	71.0	9614.0	21.8	78.2
4	10316	373.5	5.6	9523.2	332.0	30.2	10561.4	164.9	71.9	11029.8	153.6	71.1	9074.2	57.8	109.1	10076.4	75.2	106.7
5	10202	503.4	8.2	9728.6	397.7	31.2	10719.9	201.8	85.3	9311.6	171.9	108.2	9973.0	68.0	135.2	10494.3	120.3	128.2
6	9947	614.3	9.6	10290.5	453.2	34.8	9451.4	242.4	133.1	9424.6	275.2	149.8	10532.4	86.8	154.9	9963.3	106.9	132.9
7	10756	622.9	11.0	10384.1	489.8	30.6	9941.3	238.9	155.4	9647.1	383.5	100.4	10406.6	108.3	194.9	9306.5	158.9	213.7
8	9953	775.1	13.7	10231.9	693.9	71.2	10600.7	305.4	151.7	9473.0	189.3	109.4	9904.6	134.7	240.5	9346.7	123.3	201.3
9	11009	744.2	15.9	9557.1	767.1	24.3	9765.1	351.4	170.1	9677.1	551.0	234.0	8650.0	123.0	278.4	9615.8	116.4	232.7
10	9969	1024.9	18.4	9450.1	626.6	25.3	9973.8	364.2	226.7	9907.3	254.9	103.9	10030.5	111.2	305.1	9736.4	264.3	262.6
11	10652	965.0	20.6	10756.4	876.8	108.6	9956.9	447.0	229.9	9399.4	269.3	274.7	10286.9	160.5	264.2	9989.3	264.9	262.9
12	11143	1187.6	21.7	10129.0	1198.7	58.7	9556.2	453.0	251.9	11018.5	670.8	300.4	10022.9	171.0	358.4	10335.2	111.0	321.3
13	9306	1361.3	24.8	10044.2	1073.9	38.2	9777.7	449.3	276.3	10053.9	662.8	150.6	10078.7	190.9	399.9	9782.7	350.5	462.0
14	9861	1243.0	26.6	9939.6	1047.1	88.0	9985.3	527.1	276.6	9957.9	667.5	295.0	10215.8	193.9	407.2	9443.1	342.6	345.6
15	9933	1300.2	26.8	9402.8	1442.7	96.7	9793.1	561.4	319.5	10247.8	438.3	238.3	9801.7	243.6	434.4	10303.6	298.2	392.0
16	10318	1476.7	29.1	10306.0	1245.4	38.9	10556.7	615.5	348.1	10018.7	865.1	394.5	10655.0	223.0	481.6	10137.7	278.0	448.6
17	9858	1452.6	30.5	9891.4	1348.5	58.7	9759.5	733.5	353.7	9934.0	709.2	292.7	10235.2	252.9	523.6	10578.7	276.3	511.9
18	8672	1543.8	33.9	9908.6	1474.4	128.9	9783.4	636.6	383.3	10738.4	1242.0	341.7	9378.7	261.2	575.0	9158.8	199.6	539.5
19	8780	1428.1	35.0	10466.7	1140.8	74.0	10348.4	724.6	416.0	9891.5	1269.0	208.4	10690.8	265.7	573.7	10043.7	115.8	643.1
20	10660	1397.3	31.5	10410.9	1746.4	232.3	9471.8	792.0	436.9	9358.2	907.3	270.1	10602.2	307.0	582.0	10676.7	315.1	631.8

**Fig 2:**
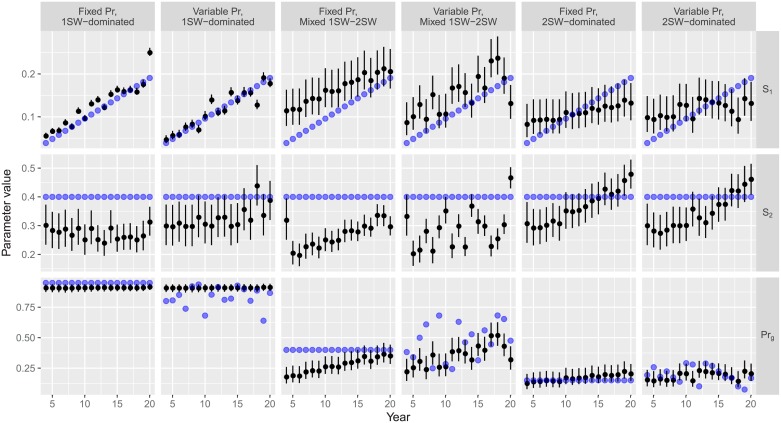
Yearly estimated *S*_1_, *S*_2_, and *P*_*r*_ values in the six scenarios. True values are denoted by blue circles, black circles show median estimates, error bars indicate the 25% and 75% quantiles.

**Fig 3:**
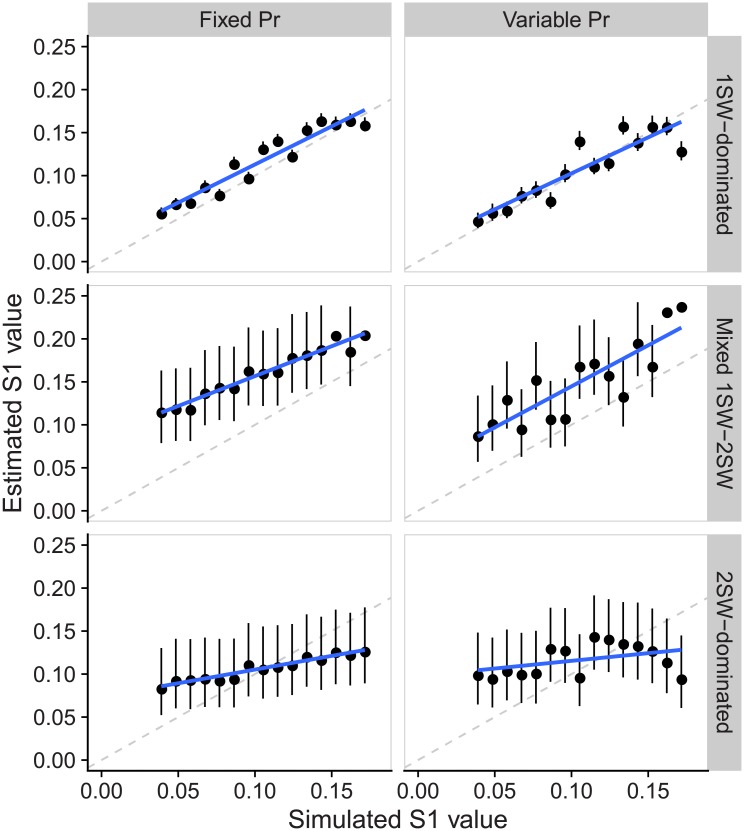
Comparison of estimated and true *S*_1_ values in the six scenarios. True *S*_1_ values are deterministic, black circles show median *S*_1_ estimates, error bars indicate the 25% and 75% quantiles, while the blue line denotes a linear model fit of the medians. The one-to-one relationship is shown by the gray dashed line.

All scenarios resulted in plausible estimates of *S*_1_ (i.e. within biologically reasonable bounds) albeit with considerable associated uncertainty, and in some cases biased with respect to the underlying simulated values (Fig 2). All but the two 2SW-dominated scenarios were able to track the temporal increase in *S*_1_ (Fig 3). Our model overestimated *S*_1_, on average, by 17.6% in the 1SW-dominated, fixed *P*_*r*_ scenario and underestimated it by -7.4% in 1SW-dominated, variable *P*_*r*_ scenario. In both Mixed 1SW-2SW scenarios, our model overestimated *S*_1_ by an even larger amount (62% and 43% on average in fixed and variable *P*_*r*_ scenarios, respectively), while the bias in *S*_1_ estimates was positive in early years and negative on latter years in the 2SW-dominated scenario given the this model was not able to track the temporal trend in *S*_1_ estimates (Table 3, Fig 3).

**Table 3:** Bias of estimates (shown as percentage difference) for estimated *S*_1_, *S*_2_, and *P*_*r*_ values across years and scenarios.

	Fixed *P*_*r*_ 1SW-dominated			Variable *P*_*r*_ 1SW-dominated			Fixed *P*_*r*_ Mixed 1SW-2SW			Variable *P*_*r*_ Mixed 1SW-2SW			Fixed *P*_*r*_ 2SW-dominated			Variable *P*_*r*_ 2SW-dominated		
Year	*S*_1_	*S*_2_	*P*_*r*_	*S*_1_	*S*_2_	*P*_*r*_	*S*_1_	*S*_2_	*P*_*r*_	*S*_1_	*S*_2_	*P*_*r*_	*S*_1_	*S*_2_	*P*_*r*_	*S*_1_	*S*_2_	*P*_*r*_
4	44.9	-24.3	-4.0	22.3	-24.9	13.8	195.8	-19.9	-54.4	124.9	-16.6	-41.3	114.7	-22.8	-13.9	155.2	-24.6	-18.5
5	39.0	-28.8	-4.0	18.3	-25.5	13.1	145.5	-48.5	-51.8	109.8	-49.0	-24.2	91.8	-26.6	-5.3	96.2	-29.3	-42.0
6	18.7	-30.3	-4.0	3.4	-22.4	7.0	104.2	-50.4	-52.6	124.2	-45.8	-38.0	61.9	-26.4	-3.7	79.8	-31.2	-8.3
7	29.2	-27.6	-4.2	14.6	-25.2	23.4	103.9	-42.8	-44.2	41.7	-29.5	-60.2	41.3	-24.2	1.5	48.4	-28.4	-30.9
8	1.0	-32.9	-4.1	9.1	-25.2	-1.1	87.2	-40.6	-41.6	98.9	-46.7	45.6	20.9	-20.5	-1.5	31.9	-24.4	-10.9
9	32.2	-26.9	-3.8	-18.1	-17.3	-2.7	65.7	-44.0	-42.2	24.0	-26.3	-61.6	9.9	-22.9	-3.9	50.7	-24.7	53.0
10	1.5	-36.9	-3.9	6.8	-23.3	33.9	70.3	-37.0	-33.5	12.3	-11.8	-6.7	16.1	-11.7	16.8	33.5	-24.4	-28.3
11	24.8	-27.1	-3.7	33.5	-25.0	6.9	52.2	-38.8	-33.0	60.0	-43.0	60.2	0.8	-12.5	11.2	-8.3	-10.2	-46.3
12	22.7	-36.7	-3.8	-3.3	-17.7	-0.1	41.0	-37.3	-34.1	49.9	-25.0	-37.0	-5.1	-11.3	15.6	25.5	-17.8	131.9
13	-1.2	-39.7	-3.6	-7.3	-17.3	12.6	43.8	-29.6	-26.1	27.0	-43.1	-19.3	-10.8	-8.0	18.1	13.4	-22.0	-21.7
14	14.9	-26.7	-3.9	18.0	-25.1	11.3	35.9	-29.0	-24.8	-0.3	-7.6	-39.1	-9.6	-3.0	30.1	1.5	-13.8	-19.3
15	14.6	-36.2	-3.7	-3.0	-23.6	-1.6	31.1	-30.0	-21.3	36.4	-21.1	38.6	-18.3	-0.9	22.2	-6.8	-6.2	-5.8
16	4.9	-34.7	-3.8	3.1	-10.5	1.7	33.9	-25.0	-12.4	10.3	-25.0	-28.4	-17.4	7.1	34.7	-16.4	-6.0	15.0
17	1.2	-34.4	-3.8	-3.0	-19.8	13.7	14.5	-26.8	-22.0	42.9	-42.6	16.9	-24.3	2.6	30.0	-29.6	5.9	3.4
18	-7.3	-36.9	-3.9	-25.1	10.0	2.7	19.4	-15.7	-13.1	38.7	-36.0	-23.4	-26.1	5.4	33.9	-44.8	5.5	46.0
19	-2.4	-33.5	-3.7	6.2	-15.7	43.4	17.8	-16.0	-7.8	5.6	-23.8	-33.6	-22.7	14.7	51.5	-20.7	11.4	204.9
20	31.7	-21.4	-3.1	-6.2	-2.6	5.7	8.4	-25.5	-11.3	-30.6	17.0	-32.0	-30.1	20.1	38.3	-30.5	15.7	24.0
mean	15.9	-31.5	-3.8	4.1	-18.3	10.8	63.0	-32.7	-31.0	45.6	-28.0	-16.7	11.4	-8.3	16.2	22.3	-13.2	14.5

With regards to estimates of the other two parameters, our model was unable to estimate *S*_2_ or *P*_*r*_ in any of the six scenarios (Fig 2). In all scenarios with variable *P*_*r*_, this variability was largely undetected by the model, but instead spuriously reflected as variability of *S*_2_ estimates (Fig 2). Regardless of the true value of *P*_*r*_, our model estimated this parameter to be around the values set in the priors, while the three scenarios with variable *P*_*r*_ resulted in highly variable estimates of *S*_2_. Furthermore, with an increasing proportion of 2SW returns in the simulation, the model spuriously assigned the temporal increase in *S*_1_ to the estimates of *S*_2_, and to a lesser extent also to the estimates of *P*_*r*_ as well (Fig 2).

## Discussion

Our study provides evidence that a simple maturation schedule matrix model, using a sample-importance-resampling algorithm without sex-specific data, is able to track temporal changes in marine survival during the first year at sea, under certain conditions, even when parameters for survival during the second year at sea and the proportion of fish returning after 1SW (*S*_2_ and *P*_*r*_, respectively) cannot be estimated properly. The ability of the model to track changes in *S*_1_ diminished with increasing relative abundance of 2SW returns in comparison of 1SW returns. While being able to estimate both *S*_2_ and *P*_*r*_ would ideally be desirable, obtaining reasonable estimates of *S*_1_ using only adult return data, is useful both as a means of estimating absolute survival as well as capturing temporal trends in survival within a given population. When absolute estimates of smolt abundance are lacking, relative indices can be used to obtain relative estimates of marine survival, which can be useful to estimate trends in marine survival through time within populations.

Our model can, thus, serve as an informative starting point for future work on estimating temporal patterns in the marine survival, in the first year at sea, of anadromous fish species. This represents a key contribution to the study of life-history variability and population viability in fishes that migrate between fresh and salt water, given that patterns in survival tend to be of greater value in examining environmental correlates of mortality than absolute estimates *per se*.

Notwithstanding the merits of the model, a number of caveats need to be considered. First, there is a degree-of-freedom problem for estimating *S*_2_ and *P*_*r*_ parameters as the product of these two parameters is a constant, meaning that they cannot be optimized individually [19]. Second, the likelihood function is arbitrary in regard to the degree to which parameter vectors are penalized, in our case being dependent on the variance of return abundances. The cost function is flexible, and could be changed to differences in absolute differences between simulated and estimated, if these are deemed to be more appropriate.

Another limitation of our model is that time-lagged parameters (specifically *S*_1_ and *P*_*r*_ from previous years needed to estimate 2SW returns; see Eq 3) have to be included as a median value from the sample-importance-resampling routine in the previous year. As a consequence, there is no uncertainty being propagated with these estimates. This shortcoming of using a sample-importance-resampling algorithm can be addressed, however, by a modelling approach that incorporates more typical Bayesian or maximum likelihood methods. It is also possible that the ability of the model to detect changes in *S*_1_ over time is not robust to different trend directions. While in our simulations we presented scenarios in which increasing values of *S*_1_ resulted in increasing return abundances, scenarios with declining abundances in return estimates provide very similar estimates of *S*_1_, *S*_2_, *P*_*r*_ and the model was also able to accurately detect the underlying *S*_1_ trend (see S1 File).

We envisage means by which our modelling approach could be strengthened. The model could incorporate a hierarchical structure, similar to what has been done by [29], to allow for partial pooling of parameters among multiple populations. Priors in a “true” Bayesian framework could also be included. To account for the non-independent estimates of *S*_1_, *S*_2_, and *P*_*r*_, random effects could be added to allow these parameters to be estimated around a distribution of potential values. In addition to data on smolt abundance, one could incorporate a S-R relationship, based on egg production, to better inform the model. That said, given the high uncertainty associated with most S-R relationships, this approach might only have inherent value when smolt data are unavailable. As mentioned previously, the model could be improved by incorporating sex-specific data, thus enabling the estimation of *S*_2_ and *P*_*r*_ (which was not possible with out model), yet this introduces the issue of accurate sexing and additional data requirements (see [17, 19]).

The estimates of *P*_*r*_ and *S*_2_ are strongly influenced by their priors; appropriate priors for these two parameters are crucial for the accurate estimation of *S*_1_. Adequately informative priors provides an alternative to the use of sex-specific return data, enabling the application of the maturity-schedule method presented here to wild populations of Atlantic salmon. While these priors can be based on expert knowledge, it might prove difficult to create priors that are grounded in empirical estimates of *S*_2_ and *P*_*r*_.

In conclusion, the modelling approach detailed here provides a means of reliably describing trends in survival of salmon during the first year at sea, that can be applied to empirical abundance estimates, particularly for populations without a predominant 2SW component to their returns. Under some life-history scenarios, absolute estimates of *S*_1_ can be made as well. However, the approach uses a somewhat arbitrary cost function, and more importantly is not able to provide information on survival for multi-sea-winter fish nor the proportion returning as 1SW (a degrees-of-freedom problem). Thus, when fitting this model to simulated population data, the resulting survival estimates for the first winter at sea (*S*_1_) are somewhat biased, given the issues associated with estimating the proportion of fish that return to spawn after spending a single winter at sea. In addition to providing temporally reliable estimates of at-sea survival during the first year for Atlantic salmon, the benefits of our modelling approach include the articulation of life-history matrix properties, the ease with which life stages can be differentially weighted in how the cost function is structured, and the simplicity of the model.

## Supporting information

S1 File (PDF)
