# Supplementary material for: Estimating marine survival of Atlantic salmon using an inverse matrix approach
Source: PLoS One. 2020 May 19;15(5):e0232407. doi: 10.1371/journal.pone.0232407 (PMC7236976; doi:10.1371/journal.pone.0232407)
Supplement: S1 File — (PDF) [file pone.0232407.s001.pdf]

Supporting Information

for

Estimating marine survival of Atlantic salmon using an inverse matrix approach

Sebastián A. Pardo[1,*], Jeffrey A. Hutchings[1]

[1] Department of Biology, Dalhousie University, Halifax, NS, B3H 4R2, Canada
[*] Corresponding author: spardo@dal.ca

This Supporting Information includes supplementary tables and figures for the publication.

Model performance with declining returning salmon abundances

Here we evaluate model performance under the same six scenarios presented in our study but with a different time series of S_1 values, which result in declining return estimates rather than increasing return estimates (Table S1, Fig S1).

Table S1: Parameters used to simulate time series data of Atlantic salmon in the six scenarios but with alternative S_1 values which result in declining returns.

Parameter	Scenario 1<br>Fixed P_r,<br>1SW-dominated	Scenario 2<br>Variable P_r,<br>1SW-dominated	Scenario 3<br>Fixed P_r,<br>mixed 1SW-2SW	Scenario 4<br>Variable P_r,<br>mixed 1SW-2SW	Scenario 5<br>Fixed P_r,<br>2SW-dominated	Scenario 6<br>Variable P_r,<br>2SW-dominated
S_1	$seq(0.02, 0.2)$	$seq(0.02, 0.2)$	$seq(0.02, 0.2)$	$seq(0.02, 0.2)$	$seq(0.02, 0.2)$	$seq(0.02, 0.2)$
S_2	0.4	0.4	0.4	0.4	0.4	0.4
P_r	0.95	$unif(0.6, 0.95)$	0.4	$unif(0.2, 0.7)$	0.15	$unif(0.05, 0.3)$

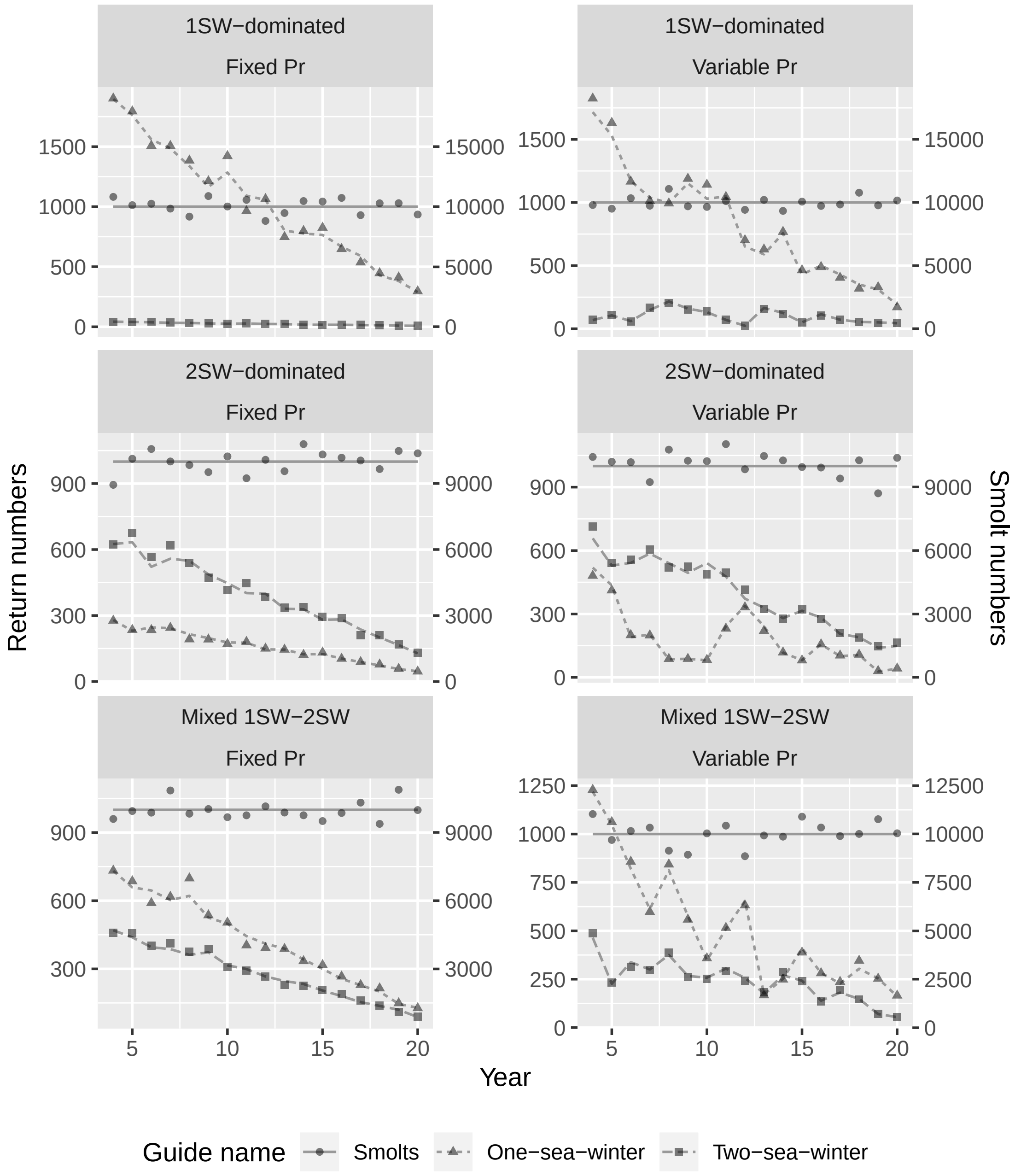

Figure S1: Simulated time series of returning adult salmon abundance in the six scenarios. The lines denote the simulated abundance estimates without observation error while the points are the same estimates including observation error.

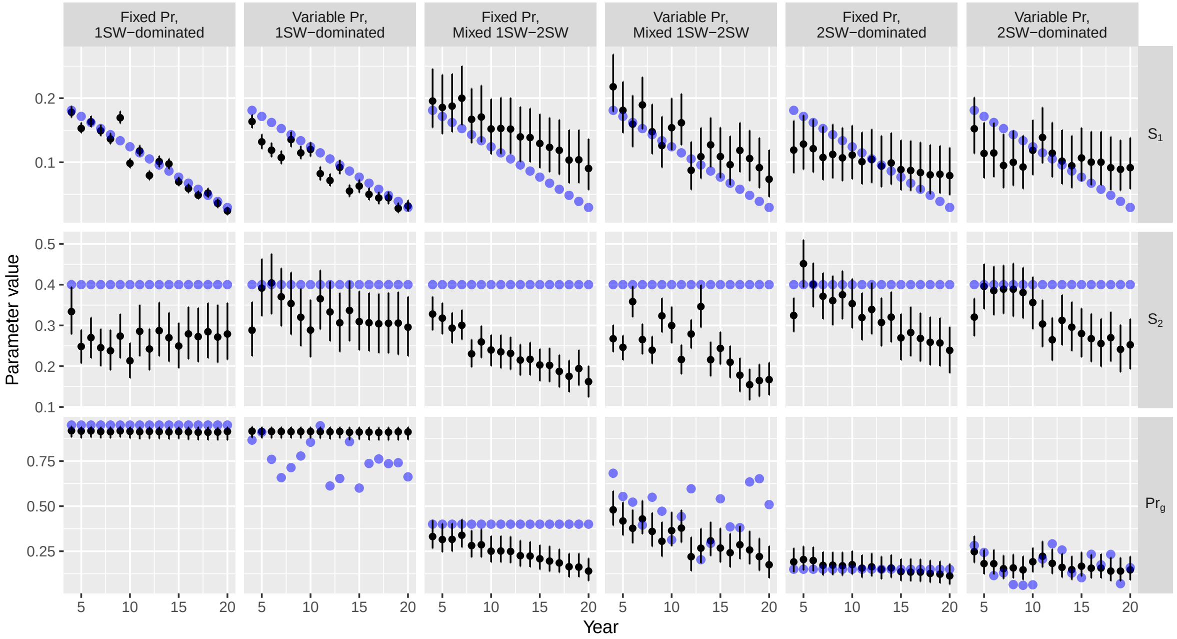

Figure S2: Yearly estimated S_1, S_2, and P_r values in the six scenarios. True values are denoted by blue circles, black circles show median estimates, error bars indicate the 25% and 75% quantiles.

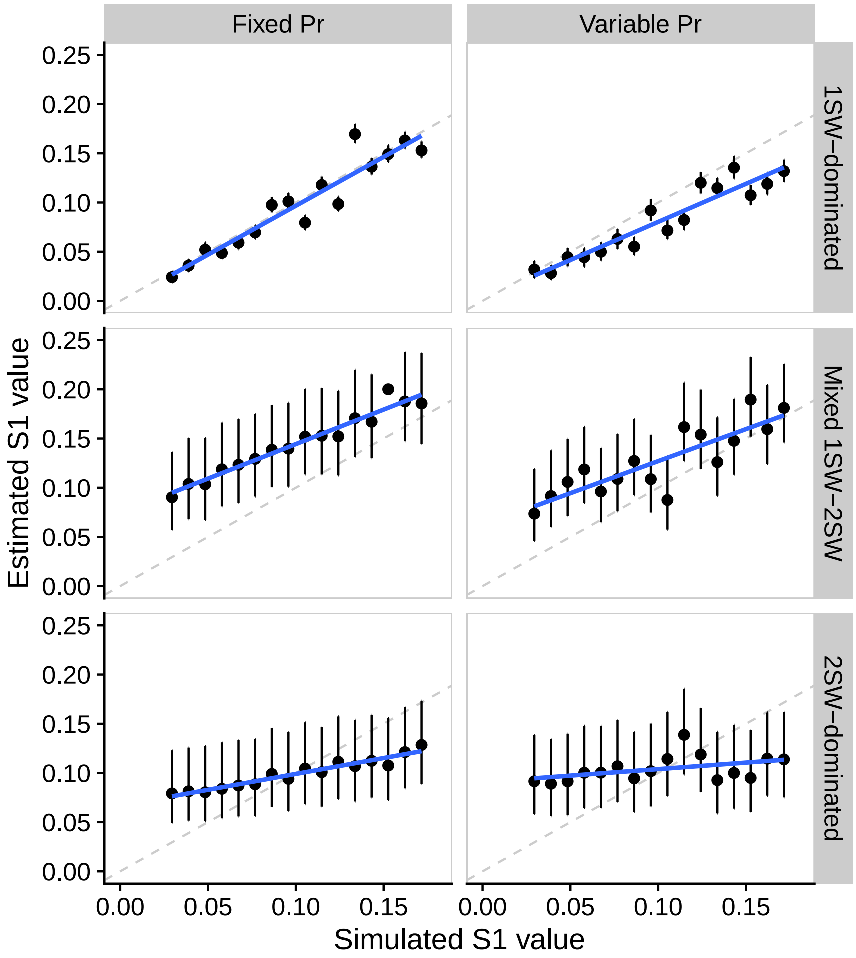

Figure S3: Comparison of estimated and true S_1 values in the six scenarios. True S_1 values are deterministic, black circles show median S_1 estimates, error bars indicate the 25% and 75% quantiles, while the blue line denotes a linear model fit of the medians. The one-to-one relationship is shown by the gray dashed line.
